# Enantioselective cellular localisation of europium(iii) coordination complexes†
†Electronic supplementary information (ESI) available: Complex synthesis and characterisation, selected cell images showing correspondence with commercial lysosomal or mitochondrial stains and spectral imaging profiles. See DOI: 10.1039/c7sc04422d


**DOI:** 10.1039/c7sc04422d

**Published:** 2017-12-06

**Authors:** Andrew T. Frawley, Holly V. Linford, Matthieu Starck, Robert Pal, David Parker

**Affiliations:** a Department of Chemistry , Durham University , South Road , Durham , DH1 3LE , UK . Email: david.parker@dur.ac.uk

## Abstract

The selective mitochondrial localisation of the *Λ* enantiomer of three different emissive europium(iii) complexes in NIH 3T3 and MCF7 cells contrasts with the behaviour of the *Δ* enantiomer, for which a predominant lysosomal localisation was observed by confocal microscopy. In each case, cell uptake occurs *via* macropinocytosis.

## Abstract 2

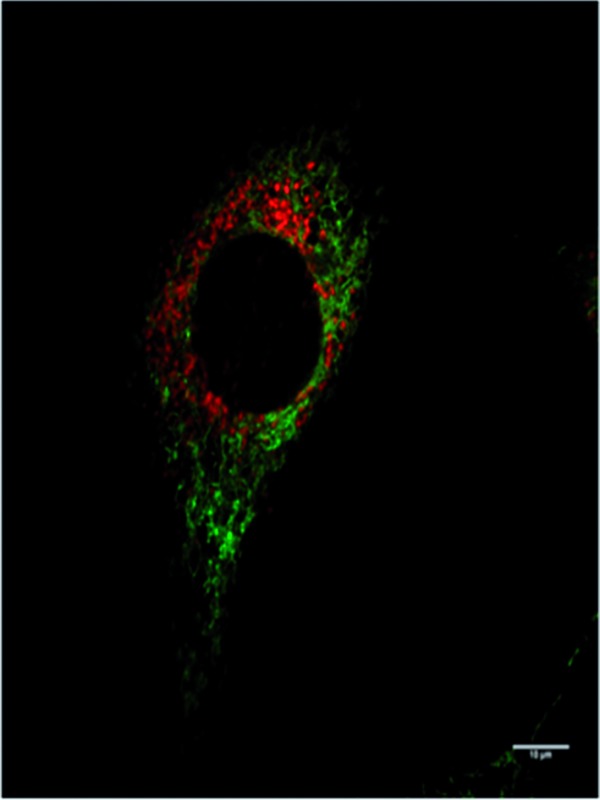
The *Λ* and *Δ* enantiomers of three luminescent europium(iii) complexes selectively stain the mitochondria and lysosomes of living cells respectively.

## Introduction

Europium(iii) complexes have been used extensively as stains for live cell fluorescence microscopy.^1^ The development of responsive probes has allowed emissive europium(iii) complexes to be used to measure the intracellular concentrations of a number of biologically relevant molecules, in addition to pH and pO_2_.^2^ Of critical importance for intracellular stains and probes, is the mechanism of uptake. In order for a complex to be used *in cellulo*, a comprehensive understanding of cell uptake and sub-cellular localisation is required to aid in the design of new probes to target particular organelles. Whilst the cell uptake mechanism of a wide range of 12-N_4_ based systems has been studied,^3^ a thorough investigation of the mechanism of internalisation of the EuroTracker™ 9-N_3_ based systems with pyridylarylalkynyl chromophores has not been undertaken. A preliminary investigation suggested that macropinocytosis was involved in the uptake of these complexes, but the presence of other competing mechanisms was not ruled out.1a,^4^

Here we report on the effect of probe chirality on the uptake process. Detailed studies of enantioselective behaviour in living cells have not been reported before. Since recognition in biological systems generally involves chiral proteins and glycoconjugates, the uptake of chiral probe species is expected to display chiral discrimination,5*a* due to differential diastereoisomeric interactions. Indeed, chiral discrimination of such complexes by sugars has been demonstrated in the resolution of the enantiomers by chiral HPLC with polysaccharide chiral stationary phases.5*b* Similarly, if the sub-cellular transport of the complexes is regulated by proteins, then variation in the localisation of the enantiomers and their rate of transfer inside the cell may also be affected.

The complexes chosen for this study, [**EuL^1–3^**], were selected as they met the following key selection criteria: solubility in water; excitation around 355 to 365 nm with a brightness of the order of 10 to 25 mM^–1^ cm^–1^ at these wavelengths; good photostability to pulsed excitation; a propensity to be taken into cells.

## Results and discussion

A cell uptake study was initially carried out using racemic [**EuL^2^**], which has already been demonstrated to be taken into cells,^6^ in order to establish the mechanism by which internalisation occurs. Secondly, the uptake and sub-cellular localisation of the enantiomers of [**EuL^1–3^**] were investigated, drawn here as the S-*Δ* enantiomers.
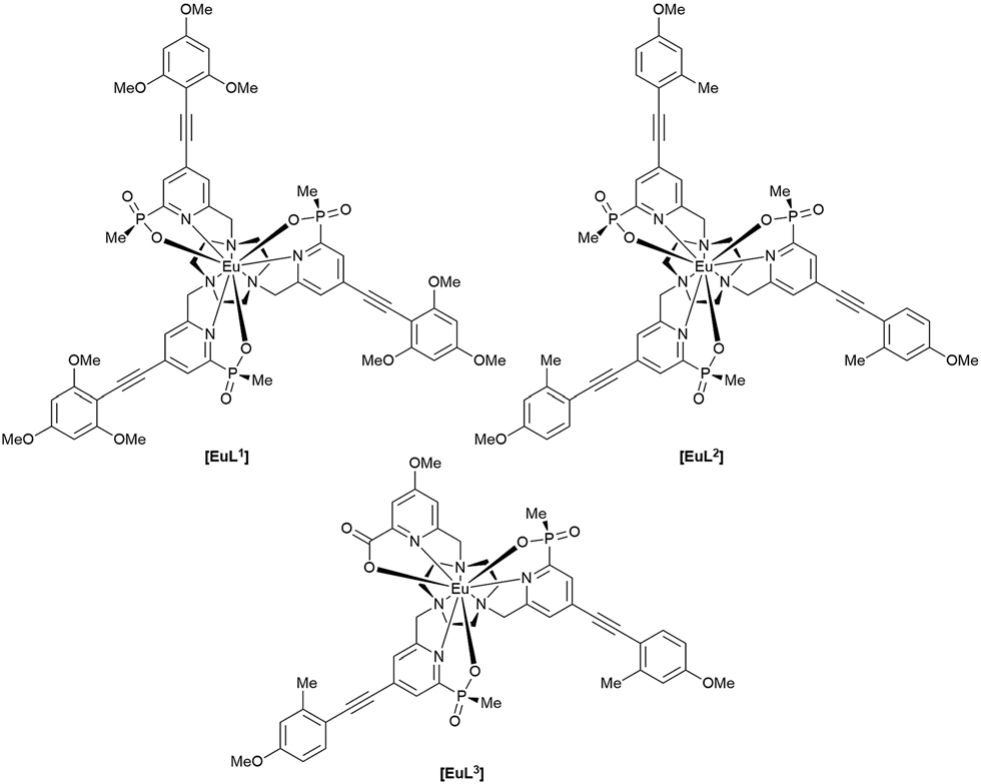


**Scheme 1:**
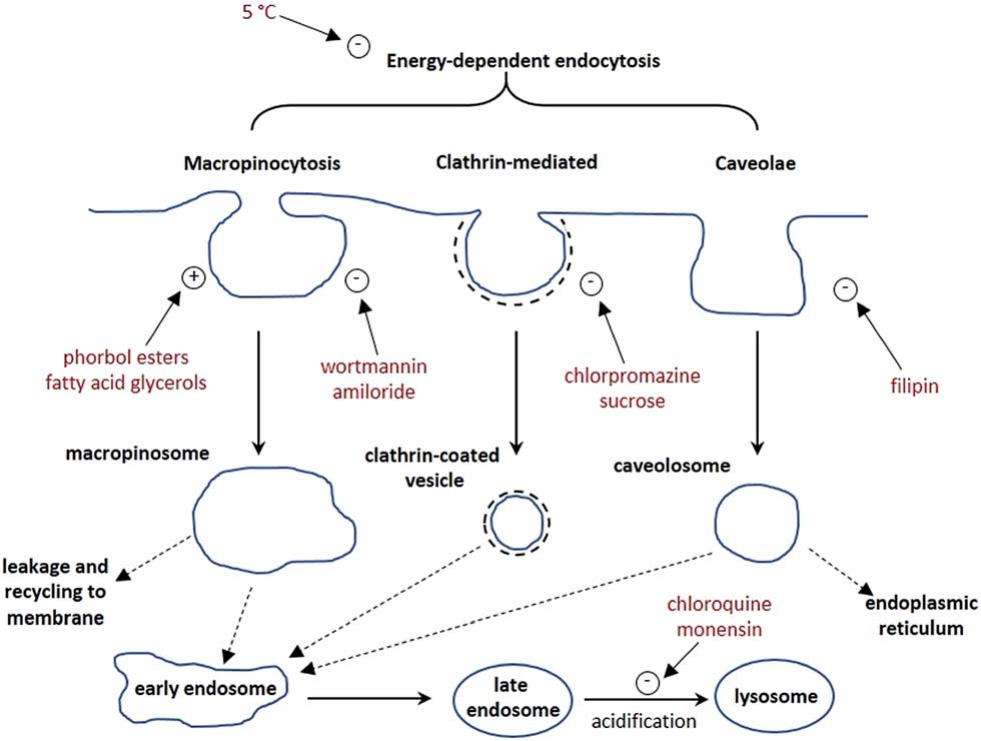
Schematic illustration of three possible endocytotic pathways for the uptake of lanthanide complexes, and the maturation of endosome to lysosomes. In red are known activators (denoted with a +) and inhibitors (–) of each process.

The synthesis of each complex used reported routes.^6^ Full synthetic details and characterisation for [**EuL^3^**] can be found in the ESI.† The cell uptake behaviour of [**EuL^2^**] was investigated in NIH-3T3 cells (mouse skin fibroblasts) in the presence of various promoters and inhibitors of macropinocytosis, clathrin-mediated endocytosis and caveolae-mediated endocytosis.^3^ For cell uptake experiments, the cells were incubated for 30 minutes in growth medium containing the promoters and inhibitors shown in Scheme 1. Cells were washed and incubated with growth medium containing [**EuL^2^**] (30 μM) and the promoters/inhibitors. After 4 hours, the coverslips were removed, washed with growth medium and mounted on glass slides.

A control sample was produced using identical procedures, without the addition of promoters or inhibitors. Laser scanning confocal microscopy images were recorded using pulsed excitation at 355 nm.^7^ The extent of complex internalisation was measured by analysis of the brightness of the acquired images. In each case, the image brightness in the 605–720 nm emission range of several perinuclear sample areas from each cell was recorded. This was repeated for multiple cells. Overall, at least 45 sample areas from multiple cells were recorded per treatment. The mean value was taken for each result and compared to the image brightness recorded using the same method for the control sample. Earlier work has shown that comparative image brightness measurements in such experiments gave values that were very similar to independent measurements of europium concentration using inductively coupled plasma mass spectrometry (ICP-MS).

**Fig. 1:**
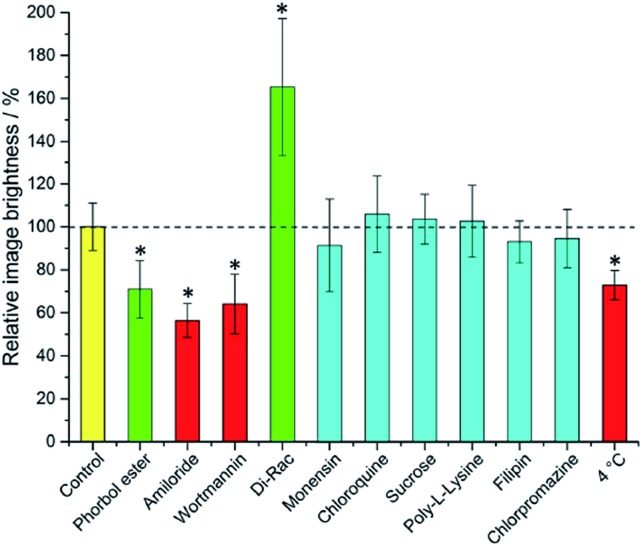
Mean relative brightness of europium(iii) emission from cells incubated with [**EuL^2^**] after treatment with promoters and inhibitors of various cell uptake mechanisms. Inhibitors (red) and promoters (green) of macropinocytosis are highlighted. Results which have statistical significance (two-tailed Student's *t*-test at 95% confidence) are marked *. Error bars represent one standard deviation from the mean.

It is immediately evident (Fig. 1) that 1,2-dipalmitoyl-rac-diacylglycerol (Di-Rac), a fatty acid glycerol and known promoter of macropinocytosis, significantly enhances the uptake of [**EuL^2^**]. After treatment of the cells with amiloride and wortmannin, both inhibitors of macropinocytosis, the internalisation of [**EuL^2^**] is reduced by about 40%, compared to the control. Similarly, for cells incubated at low temperature, the uptake of complexes is reduced, although low temperature is expected to inhibit such endocytotic pathways. Such behaviour does, however, confirm that uptake is *via* an active process. The promoters and inhibitors of other pathways do not have significant effects on the internalisation of the complex, consistent with the behaviour observed for a large series of 12-N_4_ based Eu(iii) complexes. Conspicuously, on treatment of the cells with a phorbol ester (phorbol 12-myristate 13-acetate), which is known to stimulate macropinocytosis, the brightness of the complex in the cells is reduced by approximately 30%, relative to the control. In order to assess whether the phorbol ester was quenching the [**EuL^2^**] emission and causing this anomalous result, emission spectra were recorded in cell lysate in the presence and absence of the phorbol ester. In its presence, the emission intensity was quenched by approximately 35% without much change in emission lifetime (1.11 *vs.* 1.06 ms). Such behaviour is consistent with reduced absorption efficiency or energy transfer to the metal, rather than quenching of the metal excited state. Whilst a 35% quenching effect may initially appear to account for the 30% reduced brightness in microscopy, one cannot assume that the relative concentration of the complex and the phorbol ester is the same *in cellulo* as it is *in vitro*. However, it does provide evidence that such a quenching process may account for the lower than expected emission observed for this complex by microscopy.

Taking into account all the data, it seems highly likely that macropinocytosis is the primary route of uptake of [**EuL^2^**]. Since this is a non-selective pathway, such results combined with those acquired in the preliminary study for complexes with similar structures, suggest that macropinocytosis is the primary route of uptake for this class of compounds. Previous work on 12-N_4_ complexes concluded that simultaneous passive uptake was unlikely, as complexes of different hydrophilicity (by comparison of octanol–water partition coefficients) did not show any variation in cell uptake.^3^ As the uptake of anionic, cationic and neutral complexes was affected similarly by promoters and inhibitors of macropinocytosis, it is reasonable to assume that variations in charge and hydrophilicity do not affect the uptake mechanism, across a series of otherwise structurally similar complexes.

Unlike receptor-mediated endocytosis, which is activated by binding of a specific substrate to a specific cell surface receptor, macropinocytosis is a non-selective uptake mechanism. It occurs in response to stimulation of receptor tyrosine kinases,^8^ which are *trans*-membrane proteins capable of up-regulating actin polymerisation. It is the increase of actin polymerisation that is responsible for the formation of membrane ruffling. Occasionally, the membrane ruffles fold back on themselves, forming macropinosomes. These macropinosomes are large (>0.2 μm diameter and up to 5 μm in some examples)^9^,^10^ compared to other endocytotic vesicles (*e.g.* clathrin-coated vesicles are approximately 100 nm in diameter),^11^ meaning a large volume of extracellular fluid is internalised.

It has been observed that the fate of macropinosomes varies in different cell types. In epithelial and fibroblast cells which are growth factor stimulated, macropinosomes remain relatively isolated from the endo-lysosomal system and eventually fuse back with the cell membrane.^10^ Such an observation is consistent with the need for these types of cells to constantly reorganise the plasma membrane in response to external stimuli. In contrast, in cells where degradation of extracellular material is key (such as renal cells and macrophages), macropinosomes fuse with late endosomes and lysosomes.^12^

## Effect of chirality on cell uptake and sub-cellular localisation

Although macropinocytosis is a non-specific mechanism of internalisation, the possibility of chirality dependent cell uptake and sub-cellular localisation is not precluded. For instance, favourable interaction of one enantiomer of complex with a protein or other substrate in the extracellular environment which can stimulate membrane ruffling, may result in one enantiomer being internalised more than the other. The fate of the complex once internalised may also involve transport by protein. Since proteins possess chiral binding pockets, it is possible that different sub-cellular localisations may be observed for different enantiomers.

An understanding of such behaviour is crucial in the use of emissive stains and probes in live cell imaging, both for chiral complexes and achiral complexes which may exhibit induced chirality on binding. Since complex [**EuL^2^**] has already been reported as a stain for live cell imaging,1*a* and it is simple to resolve the enantiomers by chiral HPLC, it was a logical choice for such an investigation. Additionally, parallel experiments were carried out with [**EuL^1^**] under the same conditions to ascertain whether the nature of the chromophore would affect its uptake or localisation profile.

NIH-3T3 cells were first treated with racemic [**EuL^2^**] and studied by microscopy. The complex was readily internalised by the cells and co-staining with MitoTracker Green confirmed a predominant mitochondrial localisation (*P* = 0.92) after 4 h (ESI†), consistent with the behaviour observed previously for this complex.1*a* Although mitochondria are often illustrated as ‘bean-shaped’ organelles (and often appear as such in electron micrographs where cells are fixed with heavy metals), in live cells they are dynamic, with the ability to change their shape as well as fuse and divide,^13^ and appear as a tubular network rather than as distinct organelles. The cell toxicity was checked using an MTT assay, and confirmed no evidence for impairment of mitochondrial redox activity using an MTT assay with 100 μM added complex, after a 24 h incubation.1*a*

Cell uptake experiments were repeated with enantiopure *Δ*- and *Λ*-[**EuL^2^**]. Both NIH-3T3 (mouse skin fibroblasts) and MCF7 cells (a human breast cancer cell line) were treated with complex (30 μM and 19 μM respectively) and examined by microscopy in each case after a four or 24 h incubation. No significant changes in localisation profile were evident after these different incubation times in either case. Comparison of the europium(iii) emission brightness showed that emission from the cells treated with *Λ*-[**EuL^2^**] was brighter than from the cells treated with *Δ*-[**EuL^2^**] by a factor of two in each cell line. In order to confirm that uptake of the two enantiomers was different, cells were also prepared for ICP-MS measurements. Cells dosed with complex were washed with phosphate buffered saline to remove any complex that was not internalised, before being trypsinated and harvested from the cover slip. Following digestion of the sample overnight in concentrated nitric acid, ICP-MS was used to measure the concentration of europium in the sample. This value was then used to calculate the concentration of europium in the cell sample. In the case of cells treated with [**EuL^2^**], ICP-MS analysis of a number of NIH 3T3 cell samples showed that on average there was 1.6 times more *Λ*-[**EuL^2^**] than *Δ*-[**EuL^2^**] internalised, providing further evidence that the *Λ*-enantiomer is preferentially internalised by NIH-3T3 cells.

Initial attempts to carry out the same experiments using *Δ*- and *Λ*-[**EuL^1^**] resulted in cell images that were significantly less bright that those observed for [**EuL^2^**], when cells were treated with the same concentration of complex. However, it was possible to estimate that the emission from the cells treated with *Δ*-[**EuL^1^**] was more intense by approximately 30%. ICP-MS analysis of these cell samples revealed that 1.7 times more of the *Δ*-enantiomer was internalised compared to the *Λ*-enantiomer. The reduced brightness in cells for this complex can be attributed to self-aggregation leading to quenching of the europium(iii) emission. In water, a reduction in emission lifetime is observed for [**EuL^1^**] at concentrations above 15 μM. Whilst ICP-MS data suggest that the overall concentration in the cells is not this high, it is possible that local concentrations in areas where the complex localises may be similar to this. Additionally, both enantiomers of [**EuL^1^**] were more emissive and possessed longer-lived excited states in cell growth medium than in water, suggesting that complex aggregation is suppressed to some extent in the growth medium. Repeating the cell experiments with a lower dosing of complex [**EuL^1^**] (3 μM) resulted in significantly brighter images for both enantiomers than were acquired at ten times higher loading, confirming that self-quenching was indeed occurring at higher concentration. In agreement with the more concentrated treatments, and the ICP-MS data, the cells treated with *Δ*-[**EuL^1^**] were brighter than those treated with *Λ*-[**EuL^1^**].

Spectral imaging of the complexes *in cellulo* revealed emission profiles and lifetimes that are consistent with those observed *in vitro*, confirming that the europium(iii) ion is held intact within the unperturbed ligand framework (ESI†). It is evident that in each case, one enantiomer is favourably internalised. Such behaviour might be explained if one of the enantiomers is interacting more strongly with a protein and being internalised whilst bound to protein. In order to test this hypothesis, each enantiomer of [**EuL^2^**] was titrated with bovine serum albumin (BSA, the most abundant protein in the cell growth medium). The absorption and emission spectra, as well as emission lifetimes, were monitored as a function of added BSA for each enantiomer.

**Fig. 2:**
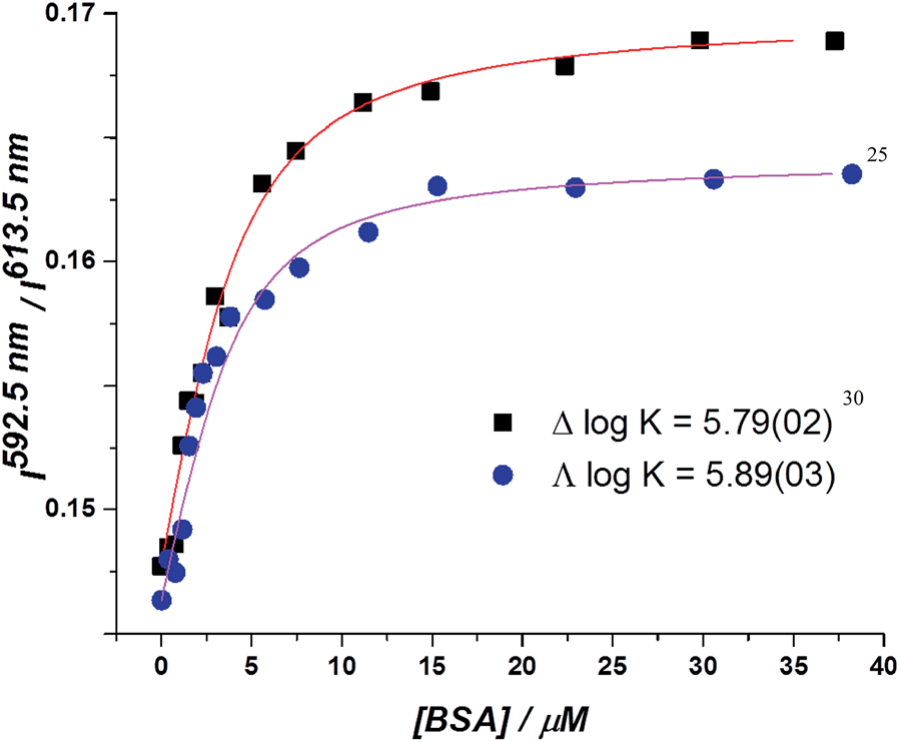
Total integrated emission intensity for *Δ*- and *Λ*-[**EuL^2^**] as a function of added BSA (4 μM complex, 0.1 M HEPES, 0.1 M NaCl, pH 7.40, 295 K, *λ*_exc_ 340 nm, 295 K); the curve shows the fit to the experimental data, derived by iterative non-linear, least squares fitting.

In the absorption spectra, the extinction coefficient of the chromophore internal charge transfer band of each enantiomer increased as BSA was added, and for *Λ*-[**EuL^2^**] a small hypsochromic shift (5 nm) of the absorption maximum was observed. Such behaviour is consistent with the chromophore experiencing a less polar environment. The total emission intensity for each enantiomer increased as a function of added BSA; a more significant change in intensity was observed for the *Λ*-enantiomer. The emission lifetime of *Λ*-[**EuL^2^**] also increased by approximately 20% as the protein concentration increased, while the lifetime of the *Δ*-enantiomer showed little variation. An estimate of the binding affinity was made by fitting the variation of an emission intensity ratio with added protein concentration; values of log *K* = 5.89 and 5.79 M^–1^ were estimated for the *Λ* and *Δ* isomers respectively (Fig. 2).

The circularly polarised luminescence (CPL) spectra of the enantiomers were also recorded as protein was added (ESI†). The CPL spectrum of *Λ*-[**EuL^2^**] increased in intensity as protein was added, consistent with the increased intensity observed in total emission. The behaviour of *Δ*-[**EuL^2^**] was more complicated. In the Δ*J* = 4 manifold, a small decrease in CPL intensity is observed, whilst a significant decrease in CPL intensity is evident in the Δ*J* = 1 and 2 manifolds. The Δ*J* = 3 manifold is largely unaffected by protein. If the complex was experiencing protein-catalysed racemisation, all manifolds would be expected to lose CPL intensity together.

The increase in emission intensity and lifetime exhibited by *Λ*-[**EuL^2^**] with added protein is consistent with the complex experiencing a less polar environment, reducing the extent of second sphere quenching by water. In addition, the modulation of luminescence intensity may be ascribed to a contribution from suppression of non-radiative deactivation pathways due to restricted motion in the protein-bound complex. Although *Δ*-[**EuL^2^**] does exhibit a small overall increase in emission intensity, the effect is not as significant as that observed for the *Λ*-enantiomer (ESI†). Such behaviour suggests that the two enantiomers are not interacting with BSA in the same manner. *Λ*-[**EuL^2^**] experiences the slightly stronger interaction with BSA, and this behaviour may tentatively be linked to why it is internalised more than *Δ*-[**EuL^2^**].

Serum albumin possesses a number of distinct binding pockets of varying structure,^14^,^15^ and is known to bind to a variety of endogenous and exogenous species, including fatty acids, metal ions, steroids, amino acids and many drugs.^16^–^19^ It is also known to bind to a variety of chelating and macrocyclic metal complexes used as contrast agents.^20^–^23^ Indeed, recent work has shown that complexes bearing pyridylarylalkynyl chromophores can bind quite strongly to serum albumin, perturbing the energy of the ICT excited state, signalled by a switching on of europium(iii) emission.^24^

It has been proposed that serum albumin proteins exhibit non-specific adsorption to cell surface membranes, acting to protect the cell membrane from physical stress, although this behaviour seems to originate from physicochemical interactions as opposed to any biological response.^25^ Studies of the interaction between BSA and lipid bilayers suggest that BSA adsorbs to the membrane at low concentrations, but causes disruption and membrane leakage at higher concentration.^26^ However, serum albumins also bind to a number of cell surface receptors, which vary depending on cell type.^27^ Binding to cell membranes allows serum albumins to deliver their cargo directly to target cells. If the *Λ*-[**EuL^2^**] enantiomer interacts more strongly with serum albumin, then it may increase the local concentration of the complex at the cell membrane, thereby increasing the probability of internalisation of this particular enantiomer.

Chiral lanthanide(III) complexes have previously been shown to exhibit differential binding to BSA.^28^ The enantiomers of complexes with tetraazatriphenylene chromophores were shown to have very different behaviour in the presence of BSA, with the *Δ* enantiomer binding to BSA with a 1 : 1 binding constant of log *K* = 5.1, while the *Λ*-enantiomer formed various low affinity adducts. Indeed, for the *Δ*-enantiomer, there was a significant change in CPL spectral form, consistent with a change in complex helicity upon binding.^29^ Whilst such significant variation in behaviour is not exhibited by [**EuL^2^**], there is a precedent for enantioselective protein binding with emissive europium(iii) complexes.

**Fig. 3:**
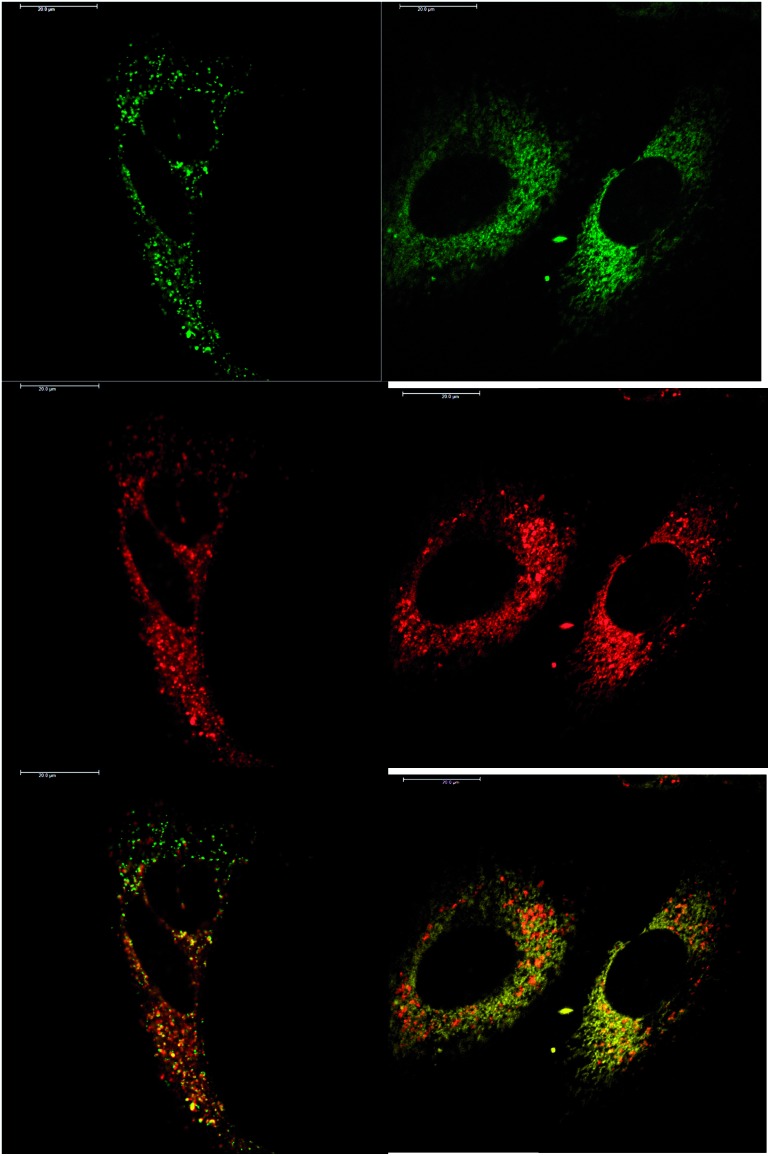
LSCM images of NIH-3T3 cells treated with *Λ*-[**EuL^2^**] (right) and *Δ*-[**EuL^2^**] (left) showing the predominant mitochondrial (*P* = 0.71) and lysosomal (*P* = 0.77) localisation profiles respectively (red for Eu emission, green for Mitotracker Green and LysoTracker Green) (24 h, 20 μM complex, *λ*_exc_ 355 nm, *λ*_em_ 605–720 nm; for tracker dyes: 0.2 μM, *λ*_exc_ 488 nm, *λ*_em_ 500–530 or 530 to 550 nm). Scale bar = 20 μm.

In addition to studying the relative uptake of the enantiomers of [**EuL^1^**] and [**EuL^2^**], the sub-cellular localisation of the enantiomers was also investigated in NIH 3T3 and MCF-7 cells. As before, the cells were incubated with enantiopure complex and imaged by laser scanning confocal microscopy. The enantiomers of [**EuL^2^**] clearly demonstrated quite different localisation profiles, with no major difference in this aspect at 4, 8 and 24 h. The *Λ*-enantiomer exhibited a predominant mitochondrial localisation revealed by the appearance of tubular networks, (Fig. 3) while the *Δ*-enantiomer exhibited a more lysosomal profile seen as circular dots, notably in the perinuclear region. Both enantiomers also produced some weak more diffuse emission from the cytosol. Co-localisation studies with MitoTracker Green and LysoTracker Green confirmed these assumptions. The estimated *P* values indicating the degree of co-localisation were 0.77 for the *Δ* complex with LTG, compared to 0.38 with MTG. For the *Λ* enantiomer, *P* values were 0.29 for LTG and 0.71 with MTG.

In MCF-7 cells the localisation profile of [**EuL^2^**] was also different for the two enantioemrs, and did not change significantly with time. At 4 h, the *Δ*-[**EuL^2^**] isomer gave *P* = 0.65 for correspondence with Lysotracker Green and *P* = 0.38 with MTG; and for the *Λ* enantiomer, *P* = 0.29 with LTG and *P* = 0.73 with MTG. Such behaviour is remarkably similar to that found in NIH 3T3 cells, (ESI Fig. S9–S12†).

After a 24 h incubation, the cells were still alive and exhibiting normal cell cycles. Indeed, it is possible to see a cell in the telophase state of mitosis (left hand panel: Fig. 3), where the nuclear membranes have reformed, and the cell is about to undergo cytokinesis, where the cell membrane separates to form two distinct cells. Similar behaviour was observed for [**EuL^1^**], with the *Λ*-enantiomer exhibiting predominant localisation in the mitochondrial network after 24 h, in contrast to the lysosomal localisation of the *Δ*-enantiomer. In order to quantify the absence of significant cellular toxicity under these conditions, an ImageCytometry viability and vitality analysis was performed on the separated enantiomers of [**EuL^2^**] in NIH 3T3 cells. This analysis (ESI†) indicated 96.3% and 95.2% cell viability at 100 μM concentrations of the *Λ* and *Δ* complexes respectively, for a 24 h loading.

**Fig. 4:**
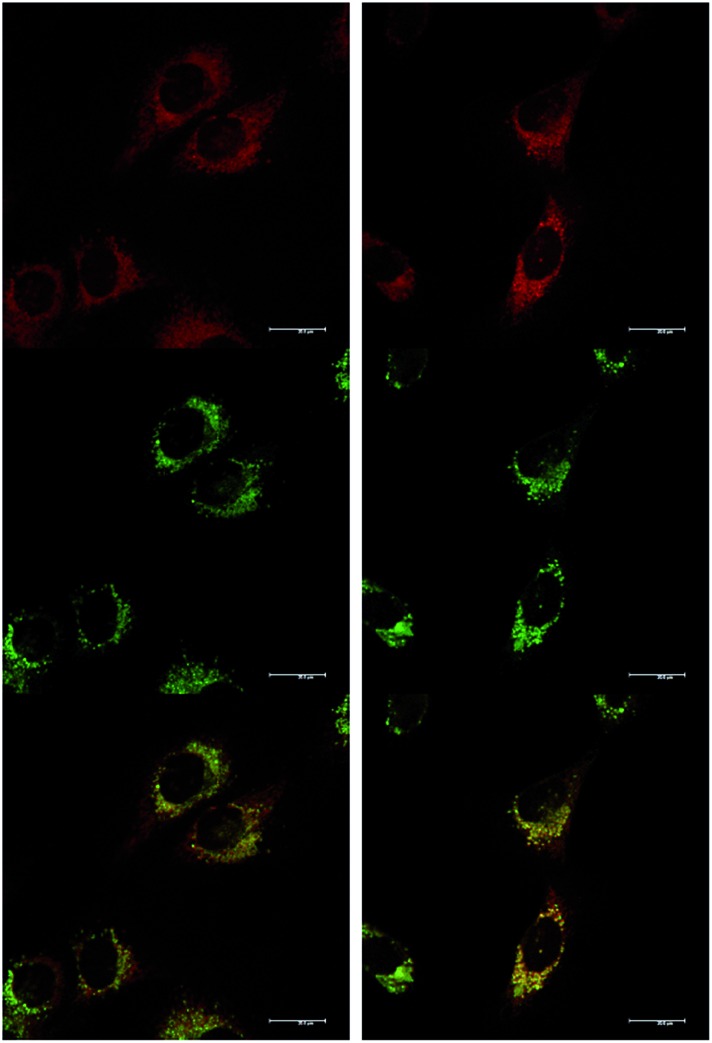
LSCM images of NIH-3T3 cells treated with *Λ*-(left) and *Δ*-(right) [**EuL^1^**] (top, 24 h, 3 μM, *λ*_exc_ 355 nm, *λ*_em_ 605–720 nm); LysoTracker Green (0.2 μM, middle, *λ*_exc_ 488 nm, *λ*_em_ 530–550 nm); and RGB merged image (bottom) (*P*_*Λ*_ = 0.65, *P*_*Δ*_ = 0.79). Scale bar = 20 μm.

At an initial complex loading concentration of 30 μM, the images of [**EuL^1^**] were not particularly bright due to a self-quenching effect. Therefore, the experiments were repeated using much lower concentrations of complex (3 μM). Additionally, co-staining with LysoTracker Green allowed comparison of the localisation profiles of both enantiomers of [**EuL^1^**] to a species known to localise to the lysosomes (Fig. 4). It is evident that the localisation profiles of the enantiomers of [**EuL^1^**] are retained, even at much lower dosing concentrations. Again, the *Λ*-enantiomer appeared to stain the mitochondria while the *Δ*-enantiomer gave predominantly lysosomal staining. These profiles were confirmed by co-staining with LysoTracker Green. The localisation of *Δ*-[**EuL^1^**] very closely matched that of LysoTracker Green, with the RGB colour merge showing almost exact co-localisation in spherical organelles concentrated around the nucleus. In contrast, the profiles for LysoTracker Green and *Λ*-[**EuL^1^**] show weaker correlation, with the complex emission appearing much more diffuse than the lysosomal localisation of the BODIPY-based stain.

Given that the different enantiomers of [**EuL^1^**] and [**EuL^2^**] localise in both the mitochondrial network and the lysosomes, it is perhaps surprising that this cannot be observed in the images acquired of cells treated with racemic complex. For example, in Fig. 2, the localisation of the racemic [**EuL^2^**] appears distinctly mitochondrial. However, it should be noted that those images were recorded after 4 hours incubation with complex, rather than after 24 hours. It is possible, therefore, that both enantiomers initially localise in the mitochondria, but that the *Δ*-enantiomer moves to the lysosomes faster than the *Λ*-enantiomer. Such time-dependent localisation behaviour has been observed previously for racemic complexes bearing both pyridylarylalkynyl chromophores and azaxanthones.^3^,^4^

**Fig. 5:**
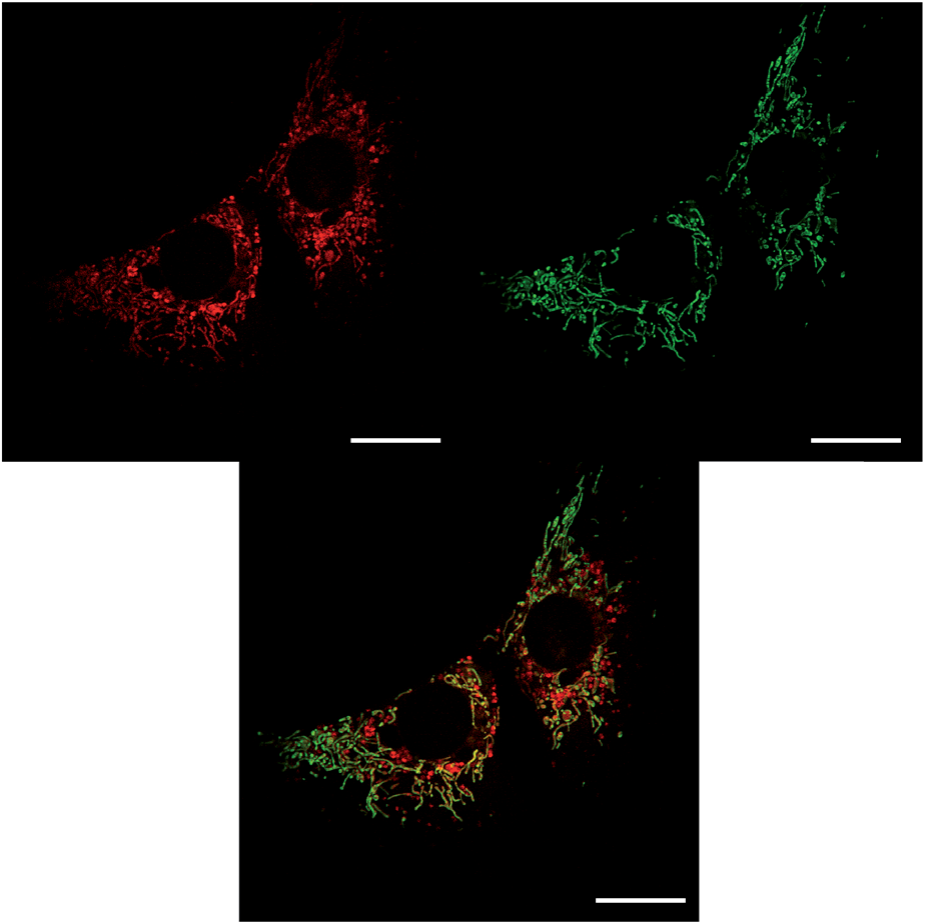
LSCM images of NIH-3T3 cells treated with *Λ*-[**EuL^3^**] and MitoTracker Green showing both mitochondrial (major) and lysosomal localisation (left, 8 h, 26 μM, *λ*_exc_ 355 nm, *λ*_em_ 605–720 nm), MitoTracker Green (right, 0.2 μM, *λ*_exc_ 488 nm, *λ*_em_ 500–530 nm) and RGB merged image (centre); scale bar = 20 μm.

The sub-cellular localisation behaviour of the closely-related complex [**EuL^3^**] is consistent with this hypothesis.^30^ Complex [**EuL^3^**] is structurally similar to [**EuL^2^**], with one extended chromophore arm replaced by a 4-methoxy substituted pyridine moiety. At early time points, *Λ*-[**EuL^3^**] exhibited localisation predominantly in the mitochondrial network, confirmed by co-staining with MitoTracker Green (Fig. 5). In contrast, europium(iii) emission from both the tubular networks of the mitochondria and the circular perinuclear lysosomes was evident in cells treated with *Δ*-[**EuL^3^**], suggesting both mitochondrial and lysosomal localisation.

Such behaviour is consistent with a time-dependent process. The complexes are internalised into macropinosomes which, in such fibroblast cells are isolated from the endosomal–lysosomal pathways.^10^ Therefore, direct trafficking of the *Δ*-enantiomers to the lysosomes is unlikely. A more likely hypothesis is that both enantiomers are transported to the mitochondria and the *Δ*-enantiomer is subsequently trafficked to the lysosomes, while the *Λ*-enantiomer remains in the mitochondria.

Macropinosomes are more prone to leakage of their contents owing to their irregular structure and lack of coating compared to endocytotic vesicles regulated by clathrin or caveolae. Leakage of macropinosome content has been observed in A431 epithelial cells, where fluorescent dyes used to label macropinosomes were found to be distributed throughout the cytosol.^31^ The cells were confirmed to be viable as they excluded trypan blue, a dye which stains only necrotic or apoptotic cells. Indeed, the complexes described exhibit weak emission from the cytosol, suggesting that some complex leaks from the macropinosomes.

The mechanisms of intracellular transport of exogenous molecules are largely ill-defined. It is likely that trafficking of the complexes to their sub-cellular destinations is *via* vesicular transport, but the processes that govern the destinations of different complexes are still unknown. Mitochondria are comprised of two membranes. The inner membrane is permeable only to small neutral molecules (oxygen, carbon dioxide and water) and contains sophisticated transport proteins to facilitate entry of specific molecules required for aerobic respiration, which occurs inside the mitochondria. The outer membrane is similar in composition to the cell surface membrane. Between the two membranes is the inter-membrane space that is broadly similar in composition to the cytosol. The outer membrane also contains porins, which are a family of barrel-shaped proteins through which large molecules (in some cases up to 5000 Da) can diffuse into the inter-membrane space.^32^ It is possible that the europium(iii) complexes diffuse through these porins and localise in the inter-membrane space, or that they localise to the outside of the outer mitochondrial membrane. From here, the observed localisation profiles suggest that the *Δ*-enantiomer is more quickly trafficked to the lysosomes than the *Λ*-enantiomer. Since the function of the lysosomes is to chemically degrade unwanted species using enzymes operating at acidic pH,^33^ it is possible that the *Δ*-enantiomer is less well tolerated by the cell, hence is removed to the lysosomes for degradation faster than the *Λ*-enantiomer. Alternatively, it is also possible that the *Λ*-enantiomer preferentially interacts with a species in the mitochondria, reducing its rate of transport to the lysosomes.

## Summary and conclusions

The primary mechanism of cell uptake of [**EuL^2^**] in mouse skin fibroblast cells and human breast cancer cells has been confirmed to be macropinocytosis, as determined by measuring complex uptake in the presence of inhibitors and promoters of various endocytotic pathways.

Using both microscopy and ICP-MS, differential uptake of the enantiomers of [**EuL^1^**] and [**EuL^2^**] has been observed. In the case of [**EuL^1^**], the *Δ*-enantiomer is internalised to a greater extent than the *Λ*-enantiomer, whilst for [**EuL^2^**] the reverse behaviour was observed in two different cell lines. Titrations of the enantiomers of [**EuL^2^**] with BSA showed varied interactions, with the *Λ*-enantiomer appearing to interact more strongly with BSA. This behaviour might explain why this enantiomer is internalised to a greater extent.

The self-quenching behaviour of [**EuL^1^**] has been explored, both *in vitro* and *in cellulo*. In cells dosed with 30 μM solutions of complex, emission was significantly quenched. Cells dosed with solutions that were 10× more dilute appeared much brighter, suggesting that at high concentration, the [**EuL^1^**] complex aggregates, resulting in a quenching of europium(iii) emission.

Comparison of the sub-cellular localisation profiles of enantiopure complexes shows that for both [**EuL^1^**] and [**EuL^2^**], the *Λ*-enantiomer predominantly localises in the mitochondrial network, while the *Δ*-enantiomer exhibits a predominantly lysosomal localisation after 24 h. Similar behaviour was found for the structurally related complex, [**EuL^3^**]. In this case, the *Δ*-enantiomer exhibits both mitochondrial and lysosomal localisation at early time points, consistent with a time-dependent localisation process.

The proposed trafficking pathway for these complexes mirrors that reported earlier for a wide range of cyclen-based probes,^3^ involves leakage of the complexes from the macropinosomes into the cytosol, followed by trafficking to the mitochondria. Subsequent removal to the lysosomes for degradation may occur more quickly for the *Δ*-enantiomer. Whilst the mechanisms by which the complexes are trafficked between various sub-cellular locations remain unknown, there is unquestionably chiral discrimination in the overall process.

There have been no reports of enantioselective localisation with rare earth complexes, and only two recent reports of the enantioselective cellular localisation of any emissive metal coordination complex. The two enantiomers of certain chiral ruthenium(ii) complexes exhibited slightly different localisation patterns with the *Δ*-enantiomer appearing in the cytosol, while the *Λ*-enantiomer seemed to concentrate around the nucleus of HepG2 cells (human liver cancer cells) and MDA-MB-231 breast cancer cells.^34^,^35^ Toxicity tests with these cationic complexes showed about 60–70% cell survival at 80 μM dose, after 24 h.

## Conflicts of interest

There are no conflicts of interest to declare.

## Supplementary Material

Supplementary information
