# Supplementary material for: Enantioselective cellular localisation of europium(iii) coordination complexes
Source: Chem Sci. 2017 Dec 6;9(4):1042–9. doi: 10.1039/c7sc04422d (PMC5883863; doi:10.1039/c7sc04422d)
Supplement: Supplementary file 1 [file SC-009-C7SC04422D-s001.pdf]

Electronic Supplementary Material (ESI) for Chemical Science.
This journal is © The Royal Society of Chemistry 2017

Enantioselective cellular localisation of europium(III) coordination complexes

Andrew T. Frawley, Holly V. Linford, Matthieu Starck, Robert Pal and David Parker*

Department of Chemistry, Durham University, South Road, Durham DH1 3LE, UK

1. Experimental and methods

2. Summary of Figures & Schemes

Scheme S1 Synthesis of **[EuL[3]]**.

Figure S1 Chiral HPLC chromatograms for [Eu.L[2]]

Figure S2 LSCM images of NIH-3T3 cells treated with racemic [EuL[2]] and MitoTracker Green[TM] .

Figure S3 Spectral imaging of **[EuL[1]]** *in cellulo*.

Figure S4 Spectral imaging of **[EuL[2]]** in cell lysate and cell lysate containing phorbol 12-myristate 13-acetate showing the quenching of emission in the presence of phorbol ester.

Figure S5 Total integrated emission intensity for *Δ*- and *Λ*-**[EuL[2]]** as a function of added bovine serum albumin (BSA).

Figure S6 CPL spectra of *Δ*- and *Λ*-**[EuL[2]]** in the presence and absence of 10 equivalents of BSA.

Figure S7 LSCM images of NIH-3T3 cells treated with *Λ*-**[EuL[1]]** showing predominant mitochondrial localisation.

Figure S8 LSCM images of NIH-3T3 cells treated with *Δ*-**[EuL[1]]** showing predominant lysosomal localisation.

Figure S9-S13 LSCM images of cells treated with the Λ or Δ enantiomers of [Eu.L[2]] in MCF-7 and NIH 3T3 cells, showing predominant localisation behaviour in correspondence with either MitoTracker Green or LysoTracker Green.

Experimental Details

General Experimental Procedures

Commercially available reagents were used as received. Electrospray mass spectra were obtained on a TQD mass spectrometer equipped with an Acquity UPLC system, an electrospray ion source and an Acquity photodiode array detector (Waters Ltd., UK). Accurate masses were recorded on an LCT Premier XE mass spectrometer or a QToF Premier Mass spectrometer, both equipped with an Acquity UPLC, a lock-mass electrospray ion source and an Acquity photodiode array detector (Waters Ltd., UK). Methanol or acetonitrile were used as the carrier solvent.

HPLC analysis

Reverse phase HPLC was performed at 295 K using a Shimadzu system comprising a Degassing Unit (DGU-20A_{5R}), a Prominence Preparative Liquid Chromatography pump (LC-20AP), a Prominence UV-Vis Detector (SPD-20A) and Communications Bus Module (CBM-20A). For preparative HPLC an XBridge C18 OBD column was used (19 x 100 mm, 5 µm) with a flow rate of 17 mL/min. For analytical HPLC a Shimadzu Shim-Pack VP-ODS column was used (4.6 x 150 mm, 5 µm) with a flow rate of 2.0 mL/min. Fraction collection was performed manually.

Chiral HPLC analysis was carried out on a Perkin Elmer Series 200 system comprising a Perkin Elmer Series 200 pump, autosampler, and UV/Vis detector, using either Daicel CHIRALPAK-IC or ID columns (4.6 x 250 mm for analytical with a flow rate of 1.0 mL/min, 10 x 250 mm for preparative with a flow rate of 4.4 mL/min, all 5 µm particle size). 100% MeOH was used as the mobile phase. Fraction collection was performed manually. Data were analysed using TotalChrom 6.3.1 software.

Optical measurements

All optical analyses were carried out in quartz cuvettes with a path length of 1 cm. UV/Vis absorbance spectra were measured on an ATI Unicam UV/Vis spectrometer (Model UV2) using Vision software (version 3.33). Emission spectra were recorded

using an ISA Jobin-Yvon Spex Fluorolog-3 luminescence spectrometer using DataMax software (version 2.2.10). Lifetime measurements were carried out using a Perkin Elmer LS55 spectrometer using FL Winlab software. Quantum yields were calculated by comparison with known standards, as described in earlier papers from this laboratory. (main paper : ref. 6).

CPL spectra were recorded on a custom-built spectrometer consisting of a laser driven light source (Energetiq EQ-99 LDLS, spectral range 170 – 2100 nm) coupled to an Acton SP2150 monochromator (600 g/nm, 300 nm Blaze) that allows excitation wavelengths to be selected with a 6 nm FWHM band-pass. The collection of the emitted light was facilitated (90° angle setup) by a Lock-In Amplifier (Hinds Instruments Signaloc 2100) and Photoelastic Modulator (Hinds Instruments Series II/FS2AA). The differentiated light was focused onto an Acton SP2150 monochromator (1200 g/nm, 500 nm Blaze) equipped with a high sensitivity cooled Photo Multiplier Tube (Hamamatsu H10723-20 PhotoSensor). Red correction is embedded in the detection algorithm and was constructed using a calibrated Ocean Optics lamp. Spectra were recorded with 0.5 nm spectral intervals and 500 µs integration time, using a 5 spectral average sequence. The monochromators, PEM control unit and lock-in amplifier were interfaced with a desktop PC and controlled by LabView code.

Cell culture protocols

Cells used for microscopy studies probing the uptake mechanism were cultured as follows. Cells were grown in a 12-well plate on a sterilised (autoclaved at 121 °C for 45 min) glass coverslip in phenol red 3-containing DMEM/F12 cell growth medium (pH 7.6) supplemented with sodium pyruvate, glucose, 0.25 mM HEPES buffer and 10% fetal bovine serum. The cells were allowed to grow to 70-80% confluence at which point the medium was removed and replaced with medium containing complex. The cells were incubated at 37 °C at 5% CO_2 and 10% humidity. For co-staining experiments, MitoTracker Green (0.2 µM) was added to the medium 30 min before imaging, while LysoTracker Green (0.2 µM) was added 5 min before imaging.

Prior to imaging, the coverslips were washed with fresh medium three times and fixed to glass slides for imaging.

Cell uptake mechanism experiments using promoters and inhibitors of various uptake pathways were carried out as follows. Cells were pre-incubated with medium containing the treatment (concentrations below) for 30 min, before being incubated at 37 °C for a further 4 h in medium containing complex (30 µM). In the case of the low temperature samples, the cells were incubated at 4 °C. The control samples were prepared in an identical fashion without promoter/inhibitor treatment. Prior to imaging, the coverslips were removed, washed with fresh medium four times and fixed to glass slides.

**Treatment**	**Dose**
Phorbol 12-myristate-13-acetate	8 nM
Amiloride	30 µM
Wortmannin	3 nM
1,2,-Dipalmitoyl-*rac*-diacylglycerol	90 nM
Monensin	0.2 nM
Chloroquine	1 µM
Sucrose	0.5 mM
Poly-L-lysine	0.001% w/v
Filipin	1.5 µM
Chlorpromazine	5 µM

In each case, the following experimental parameters were used for imaging. For europium complexes: λ_{exc} = 355 nm, λ_{em} 605-720 nm; for MitoTracker Green: λ_{exc} = 488 nm, λ_{em} 500-530 nm; for LysoTracker Green: λ_{exc} = 488 or 514 nm, λ_{em} 530-550 nm.

A more detailed investigation of the cellular behaviour of each complex was conducted using mouse skin fibroblasts (NIH-3T3) and Human Brest Adenocarcinoma (MCF7) cell lines using fluorescence and laser scanning confocal microscopy. Cells were maintained in exponential growth as monolayers in F-12/DMEM (Dulbecco's Modified Eagle Medium) 1:1, that was supplemented with 10% foetal bovine serum

(FBS). Cells were grown in 75 cm^2 plastic culture flasks, with no prior surface treatment. Cultures were incubated at 37 °C, 20% average humidity and 5% (v/v) CO_2. Cells were harvested by treatment with 0.25% (v/v) trypsin solution for 5 min at 37 ºC. Cell suspensions were pelleted by centrifugation at 1000 rpm for 3 min, and were re-suspended by repeated aspiration with a sterile plastic pipette. Microscopy Cells were seeded in 12-well plates on 13mm 0.17mm thick standard glass cover-slips or un-treated iBibi 100 uL live cell channels and allowed to grow to 40% – 60% confluence, at 37 ºC in 5% CO_2. At this stage, the medium was replaced and cells were treated with complexes and co-ctains as appropriate. For imaging DMEM media (10% FBS) lacking phenol red was used from this point onwards. Following incubation, the cover-slips were washed with phosphate-buffered saline (PBS; pH 7.4), mounted on slides and the edges sealed with colourless, quick-dry nail varnish to prevent drying out of the sample.

Cell toxicity measurements were run using a ChemoMetec A/S NucleoCounter3000-Flexicyte instrument with Via1-cassette cell viability cartridge (using the cell stain Acridine Orange for cell detection, and the nucleic acid stain DAPI for detecting non-viable cells). The experiments were done in triplicate. In cellular uptake studies, the cells were seeded in 6-well plates and allowed to grow to 80% – 100% confluence, at 37 C in 5% CO_2. At this stage, the medium was replaced with medium containing the europium complexes as detailed above , and total cellular europium was determined using ICP-MS, (inductively coupled plasma mass spectrometry) by Dr. C. Ottley in the Department of Earth Sciences at Durham University. For a 24h incubation of 100 microM Delta-[Eu.L2] in NIH 3T3 cells, cell viability and vitality was 95.2%, compared to 96.3 % for the Lambda enantiomer under identical conditions, consistent with an absence of significant cellular toxicity under these conditions.

Cells used for ICP-MS studies were prepared as follows. Cells were cultured in a 6-well plate to 90% confluence. Cells were then counted (10^7 cells based on a cell volume of 4000 μm^3) and incubated with medium containing the complex before being washed three times with phosphate-buffered saline (PBS). The cells were then trypsinated and harvested and diluted to 1 mL with PBS. Concentrated nitric acid

(0.6 mL) was added and the samples were digested for 24 h at 37 °C. These digested samples were submitted for ICP-MS measurements (Dr. Chris Ottley, Department of Earth Sciences, Durham University). The samples were run against a series of Eu standards, and the measured concentration was back calculated to find the total Eu concentration present in the original counted cells.

Confocal microscopy and cell imaging

Cell images and co-localisation experiments were obtained using a Leica SP5 II microscope equipped with PhMoNa.[7 main paper] In order to achieve excitation with maximal probe emission, the microscope was coupled by an optical fibre to a coherent 355 nm CW (Nd:YAG) laser (3rd harmonic), operating at 8 mW power. A HeNe or Ar ion laser was used when commercially available organelle-specific stains (e.g. MitoTracker Green and LysoTracker Green) were used to corroborate cellular compartmentalisation. The microscope was equipped with a triple channel imaging detector, comprising two conventional PMT systems and a HyD hybrid avalanche photodiode detector. The latter part of the detection system, when operated in the BrightRed mode, is capable of improving imaging sensitivity above 550 nm by 25%, reducing signal to noise by a factor of 5. The pinhole was determined by the Airy disc size, calculated from the objective in use (HCX PL APO 63x/1.40 NA Lbd Blue), using the lowest excitation wavelength (355 nm) and was set to 0.6 Airy unit. Scanning speed was adjusted to 200 Hz in a unidirectional mode, to ensure both sufficient light exposure and enough time to collect the emitted light from the lanthanide based optical probes (1024 x 1024 frame size, a pixel size of 120 x 120 nm and depth of 0.772 μm) but sufficiently fast to prevent movement of cellular compartments due to natural homeostasis. Spectral imaging in cells was achieved using a custom-built microscope (modified Zeiss Axiovert 200M), using a Zeiss APOCHROMAT 63x/1.40 NA objective combined with a low voltage (24 V) 365 nm pulsed UV LED focused, collimated excitation source (1.2 W). For rapid spectral acquisition the microscope was equipped at the X1 port with a Peltier cooled 2D-CCD detector (Maya Pro, Ocean Optics) used in an inverse 100 Hz time gated sequence. Spectra were recorded from 400-800 nm with a resolution of 0.24 nm and averaged using a 10,000 scan duty cycle. Probe lifetimes were measured on the same microscope

platform using a novel cooled PMT detector (Hamamatsu H7155) interchangeable on the X1 port, with the application of pre-selected interference filters. Both the control and detection algorithm were written in LabView2014, where probe lifetime was determined by using a single exponential fitting algorithm to the monitored signal intensity decay.

Synthetic Procedures

The synthesis and characterisation of the europium complexes of **[EuL^1]** and **[EuL^2]** were carried out as reported in Ref. 6 (main text).

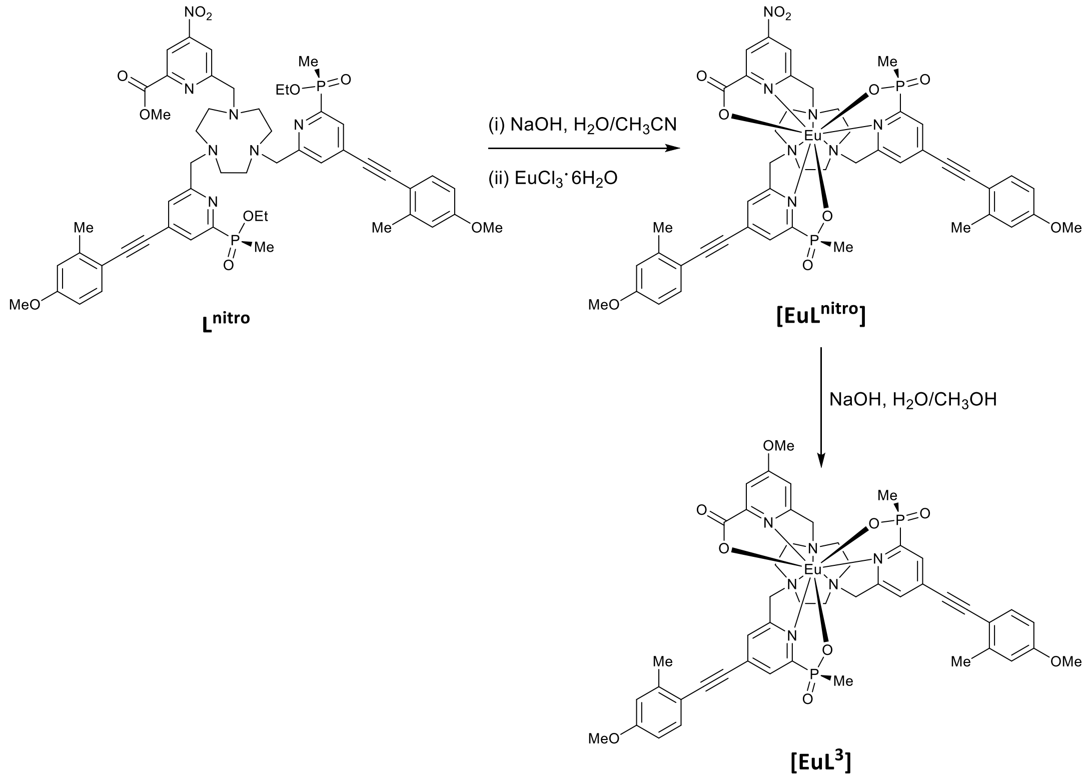

Scheme S1 Synthesis of **[EuL^3]**

The synthesis of the ligand **L^{nitro},** 6-((4,7-bis((6-(ethoxy(methyl)phosphoryl)-4-((4-methoxy-2-methylphenyl)ethynyl)pyridin-2-yl)methyl)-1,4,7-triazacyclononan-1-yl)methyl)-4-nitropicoline, is being reported elsewhere.

Europium complex of 6-((4,7-bis((6-(ethoxy(methyl)phosphoryl)-4-((4-methoxy-2-methylphenyl)ethynyl)pyridin-2-yl)methyl)-1,4,7-triazacyclononan-1-yl)methyl)-4-nitropicolinate, [EuL^{nitro}]

Ligand $\mathbf{L^{nitro}}$ (25.0 mg, 24.5 µmol) was dissolved in a solution of CH_3CN (1.5 mL), and a solution of aqueous sodium hydroxide (0.5 mL, 0.1 M) was added. The solution was stirred at room temperature and hydrolysis of ester groups was monitored by LCMS until the complete formation of the deprotected ligand: HRMS (ESI^+) *m/z* 936.3232 $[M+H]^+$ ($C_{47}H_{52}N_7O_{10}P_2$ requires 936.3251). After hydrolysis, the pH was adjusted to 7.0 by addition of an aqueous HCl solution (1 M). $EuCl_3{\cdot}6H_2O$ (9.8 mg, 26.9 µmol) was added and the solution was stirred at room temperature for 12 h. The solvent was removed under reduced pressure to yield the crude Eu(III) complex as a yellow solid which was purified by HPLC (10 to 100 % CH_3CN in 25 mM NH_4HCO_3 buffer over 10 min, t_r = 5.9 min) to give the pure europium complex **[EuL^{nitro}]** (4.6 mg, 17 %) as a yellowish powder after freeze drying; HRMS (ESI^+) *m/z* 1084.2224 $[M+H]^+$ ($C_{47}H_{49}N_7O_{10}P_2{}^{151}Eu$ requires 1084.2215); $\tau_{H2O\ (ammonium\ bicarbonate)}$ = 0.81 ms; τ_{D2O} = 1.05 ms; τ_{MeOH} = 1.15 ms; q = 0; λ_{max} = 340 nm; $\varepsilon_{340\ nm}$ = 41 000 $M^{-1}.cm^{-1}$; Φ_{H2O} = 5 %, Φ_{MeOH} = 39 %.

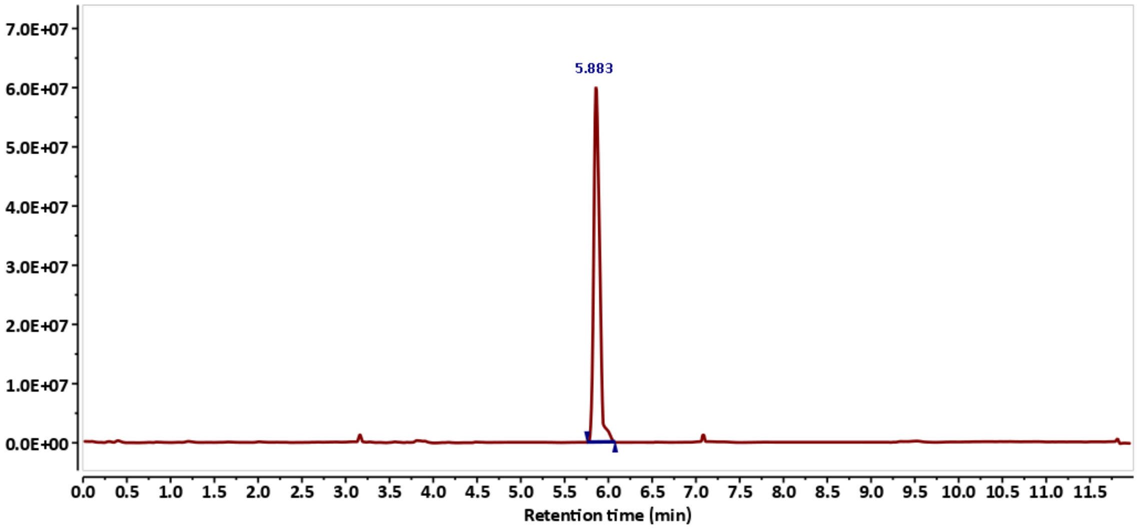

Europium complex of 6-((4,7-bis((6-(hydroxy(methyl)phosphoryl)-4-((4-methoxy-2-methylphenyl)ethynyl)pyridin-2-yl)methyl)-1,4,7-triazacyclononan-1-yl)methyl)-4-methoxypicolinate, [EuL^3]

Complex **[EuL^{nitro}]** (2 mg, 1.85 µmol) was dissolved in water/methanol (1:1, 2 mL) and the pH was adjusted to 8.0 using aqueous NaOH. The solution was stirred at room temperature for 12 hours and was directly purified by HPLC (10 to 100 % CH_3CN in 25 mM NH_4HCO_3 buffer over 10 min, t_r = 5.8 min) to give the pure to give

pure [**EuL^3**] (1.7 mg, 85 %) as a white solid. HRMS (ESI$^+$) *m/z* 1069.2463 $[M+H]^+$ ($C_{48}H_{52}N_6O_9P_2{}^{151}Eu$ requires 1069.2470); τ_{H2O} = 0.92 ms; λ_{max} = 339 nm; $\varepsilon_{339\ nm}$ = 41000 $M^{-1}.cm^{-1}$; Φ_{H2O} = 32 %, Φ_{MeOH} = 57 %.

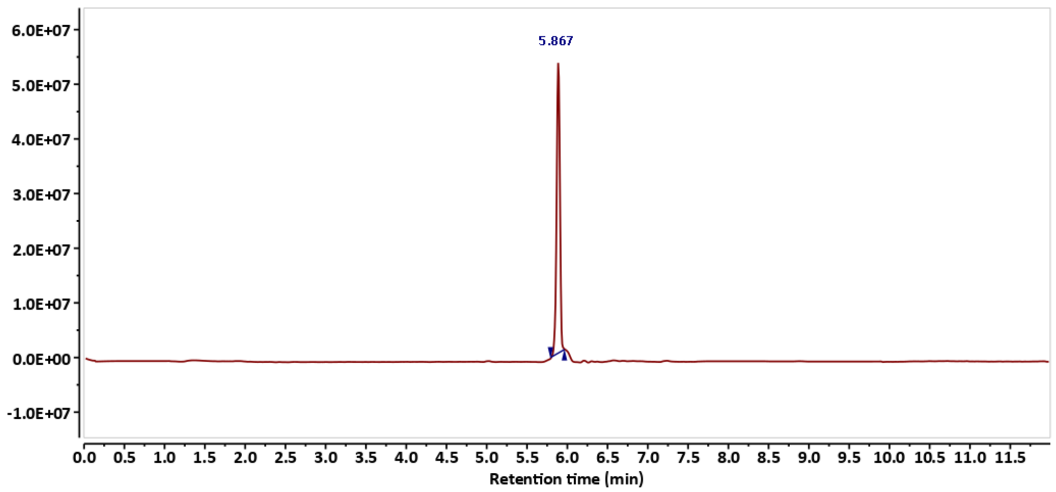

Chiral resolution of complexes **[EuL^1]** and **[EuL^2]** has been reported previously (Ref. 5, main text). Chiral separation of **[EuL^3]** was achieved using a ChiralPak ID column (10 x 250 mm, 5 µm particle size, isocratic MeOH, 4.4 mL/min); t_R = 12.9 & 33.9 min. Enantiomeric identification was achieved by comparison of the CPL spectral form to analogues whose structures have been confirmed by X-ray crystallography.

Chiral resolution of complex [EuL^2]

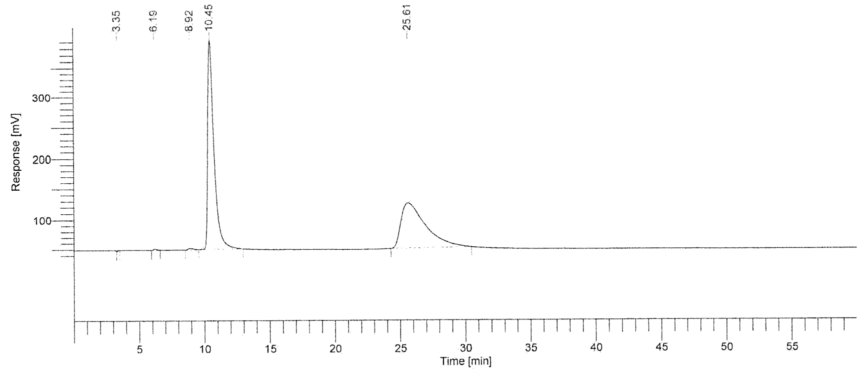

Peak #	Time [min]	Area [µV·s]	Height [µV]	Area [%]	Norm. Area [%]	BL	Area/Height [s]
4	10.449	11514078.09	342756.33	53.55	53.55	VB	33.5926
5	25.613	9986729.00	75302.03	46.45	46.45	BB	132.6223
		21500807.09	418058.36	100.00	100.00		

Chiral analytical HPLC of Δ-[EuL^2] after resolution of complex [EuL^2]

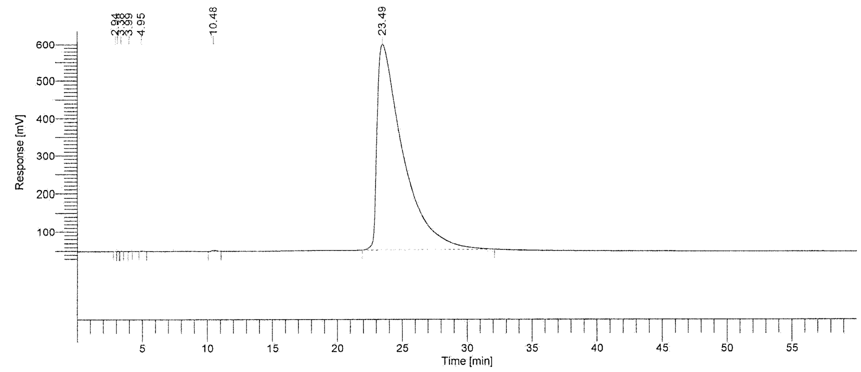

Peak #	Time [min]	Area [µV·s]	Height [µV]	Area [%]	Norm. Area [%]	BL	Area/Height [s]
7	23.492	75012888.00	547460.93	100.00	100.00	BB	137.0196
		75012888.00	547460.93	100.00	100.00		

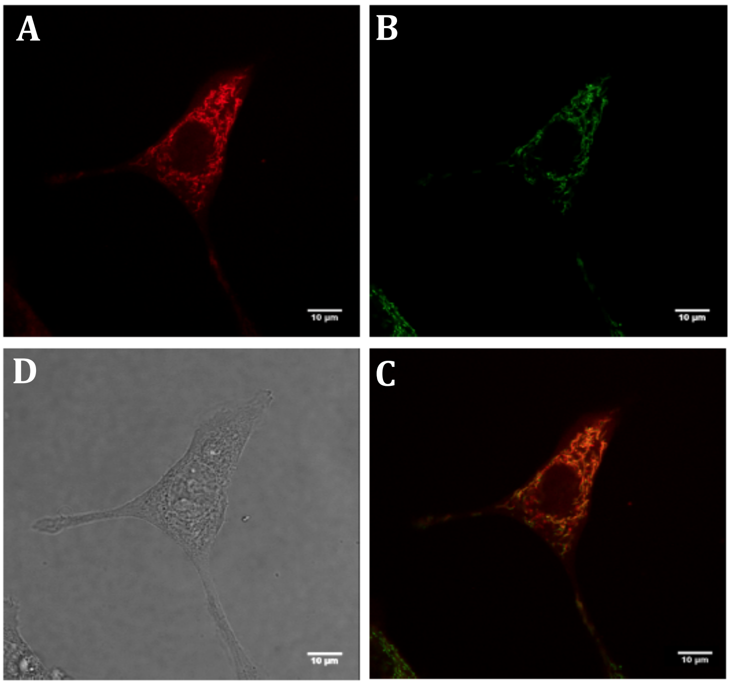

Figure S2 LSCM images of NIH-3T3 cells treated with racemic **[EuL^2]** and MitoTracker Green showing (A) mitochondrial localisation of **[EuL^2]** (4 h, 30 µM, λ_{exc} 355 nm, λ_{em} 605-720 nm); (B) MitoTracker Green (0.2 µM, λ_{exc} 488 nm, λ_{em} 500-530 nm); (C) RGB merged image showing co-localisation (P = 0.92); (D) A transmission image of the cell of interest.

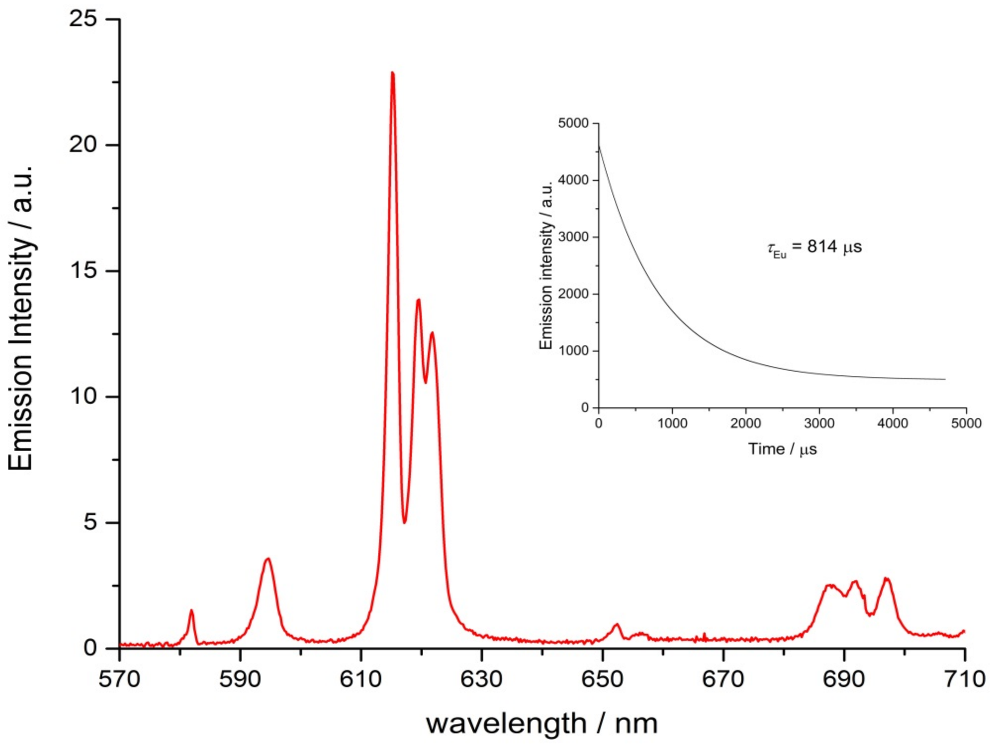

Figure S3 Spectral imaging of **[EuL^1]** *in cellulo* showing an emission profile consistent with that obtained *in vitro,* and an emission lifetime of 0.81 ms.

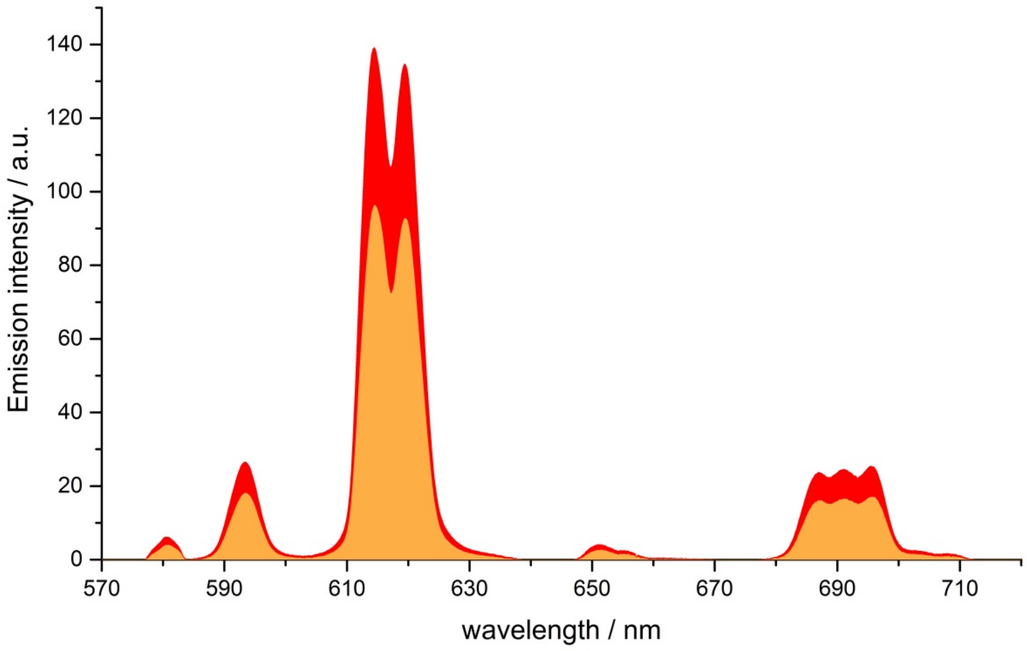

Figure S4 Spectral imaging of **[EuL^2]** in cell lysate (*red*) and cell lysate containing phorbol 12-myristate 13-acetate (*yellow*) (λ_{exc} 355 nm).

[EuL^2] vs. BSA titration

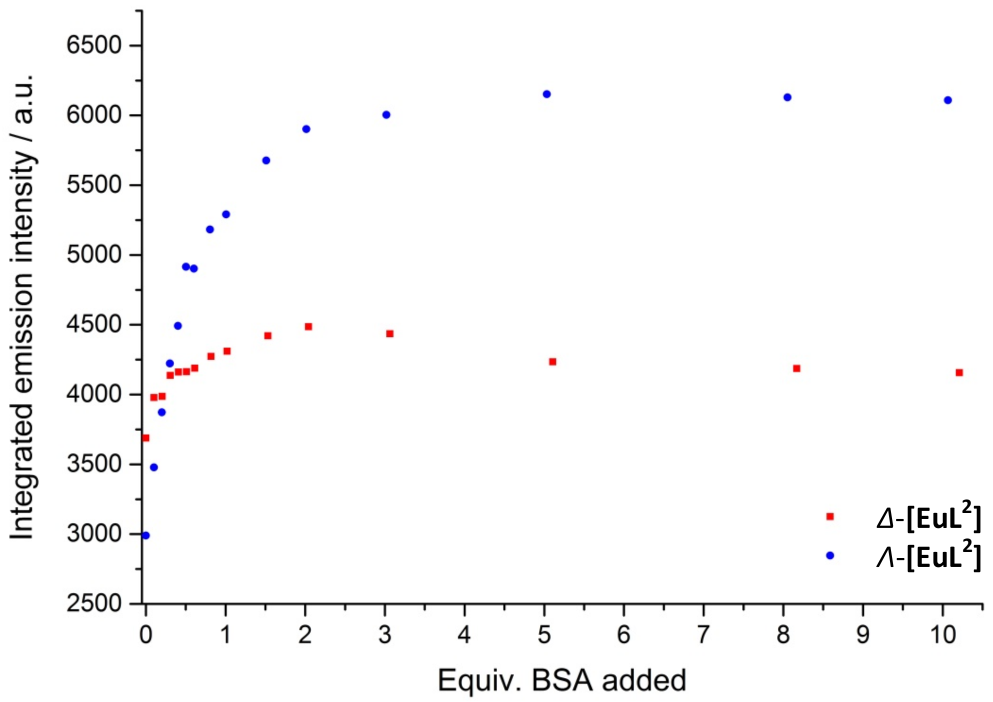

Figure S5 Total integrated emission intensity for *Δ*- and *Λ*-**[EuL^2]** as a function of added BSA (4 µM complex, 0.1 M HEPES, 0.1 M NaCl, pH 7.40, 295 K, λ_{exc} 340 nm).

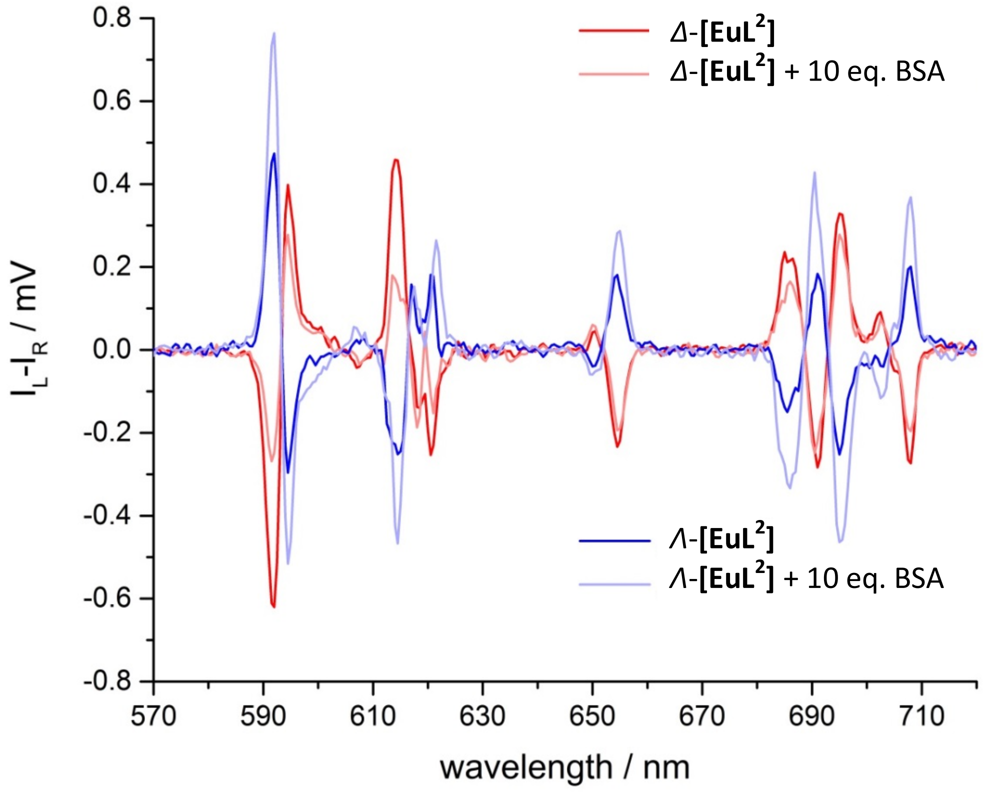

Figure S6 CPL spectra of *Δ*- and *Λ*-**[EuL^2]** in the presence and absence of 10 equivalents of BSA (4 µM complex, 0.1 M HEPES, 0.1 M NaCl, pH 7.40, 295 K, λ_{exc} 340 nm).

Enantioselective sub-cellular localisation of $[EuL^1]$

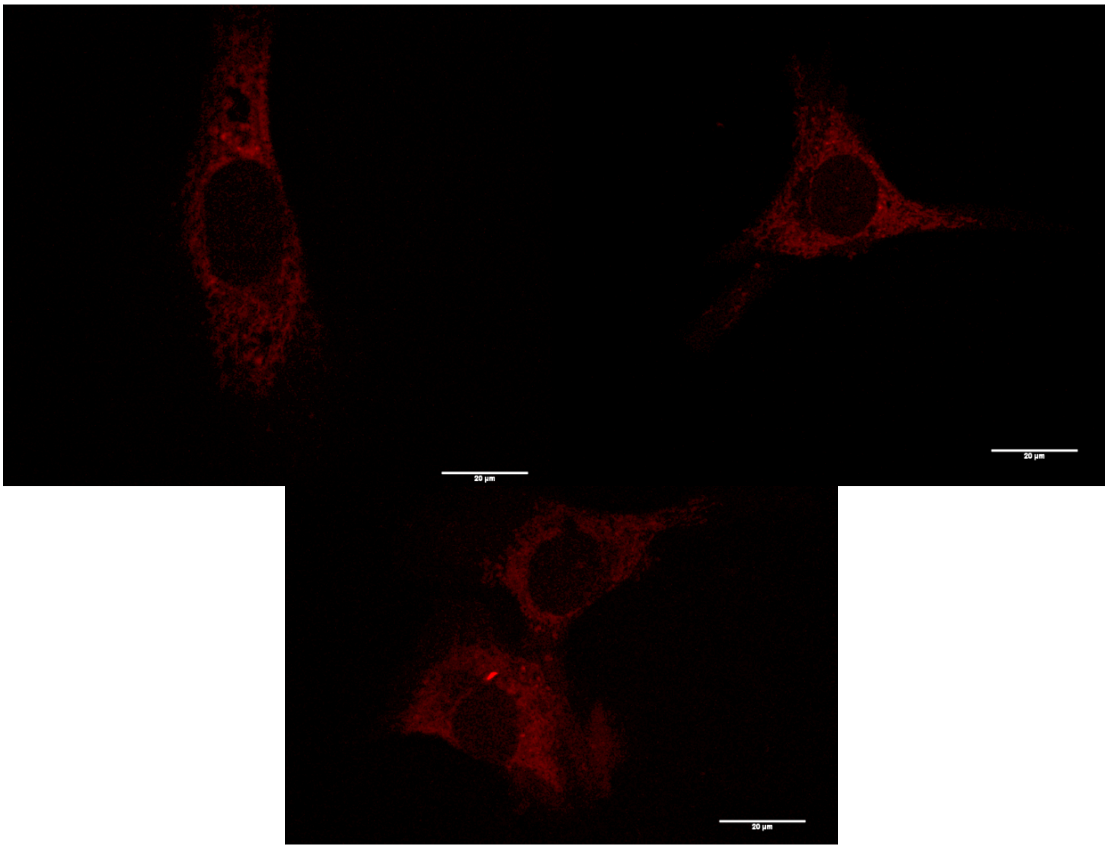

Figure S7 LSCM images of NIH-3T3 cells treated with Λ-**$[EuL^1]$** showing mitochondrial localisation (24 h, 30 µM, λ_{exc} 355 nm, λ_{em} 605-720 nm). Scale bars denote 20 µm.

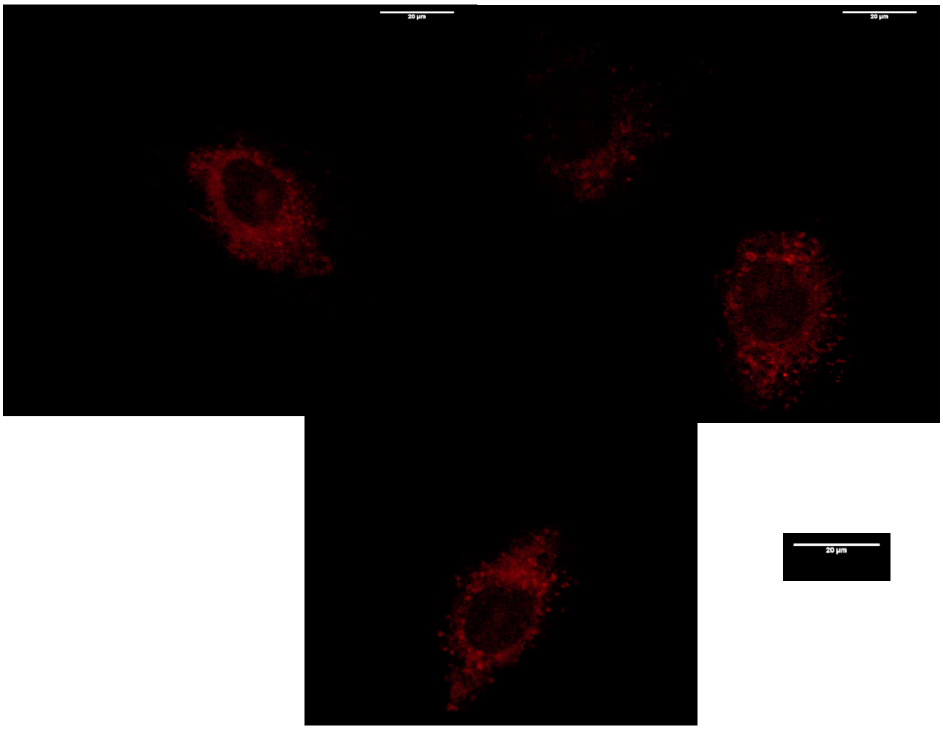

Figure S8 LSCM images of NIH-3T3 cells treated with *Δ*-**[EuL[1]]** showing predominant lysosomal localisation (24 h, 30 µM, λ_{exc} 355 nm, λ_{em} 605-720 nm). Scale bars denote 20 µm.

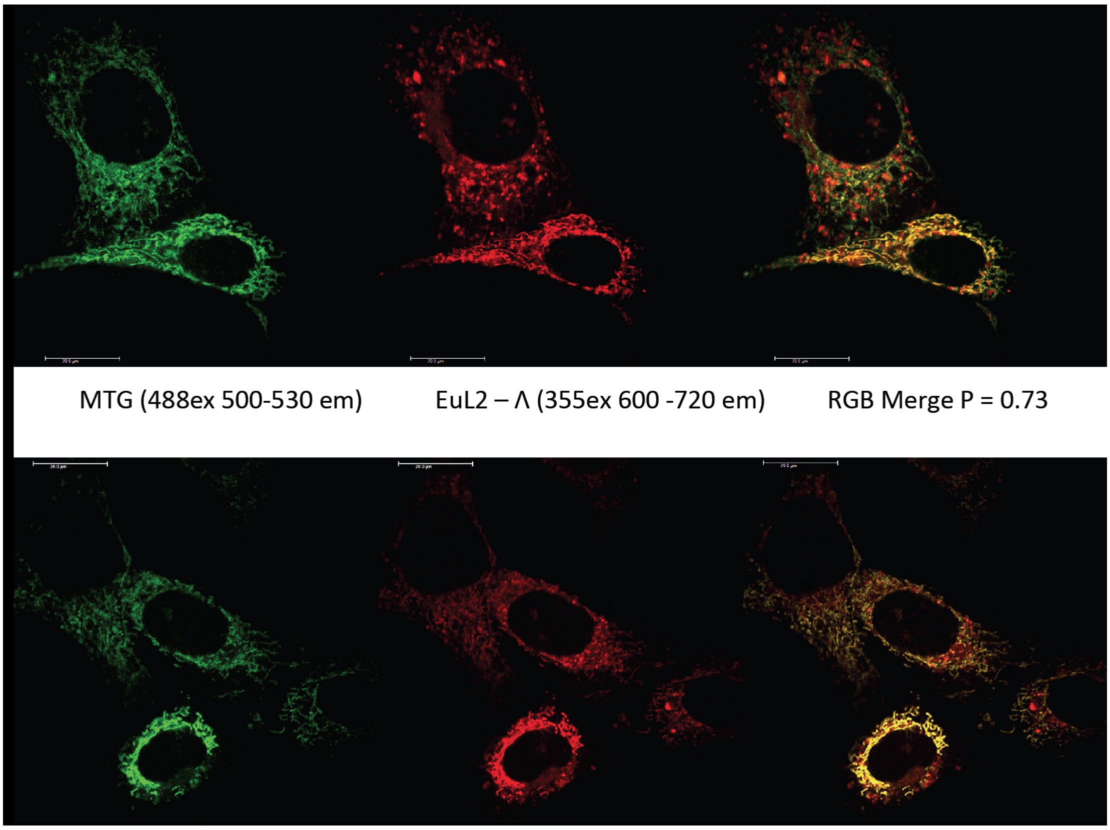

Figure S9 LSCM images of MCF-7 cells treated with *Λ*-**[EuL[2]]** showing predominant mitochondrial localisation, P = 0.73 (24 h, 19 µM: Eu , λ_{exc} 355 nm, λ_{em} 605-720 nm; MTG: λ_{exc} 488 nm, λ_{em} 500-530 nm). Scale bars denote 20 µm, (MTG = MitoTracker Green).

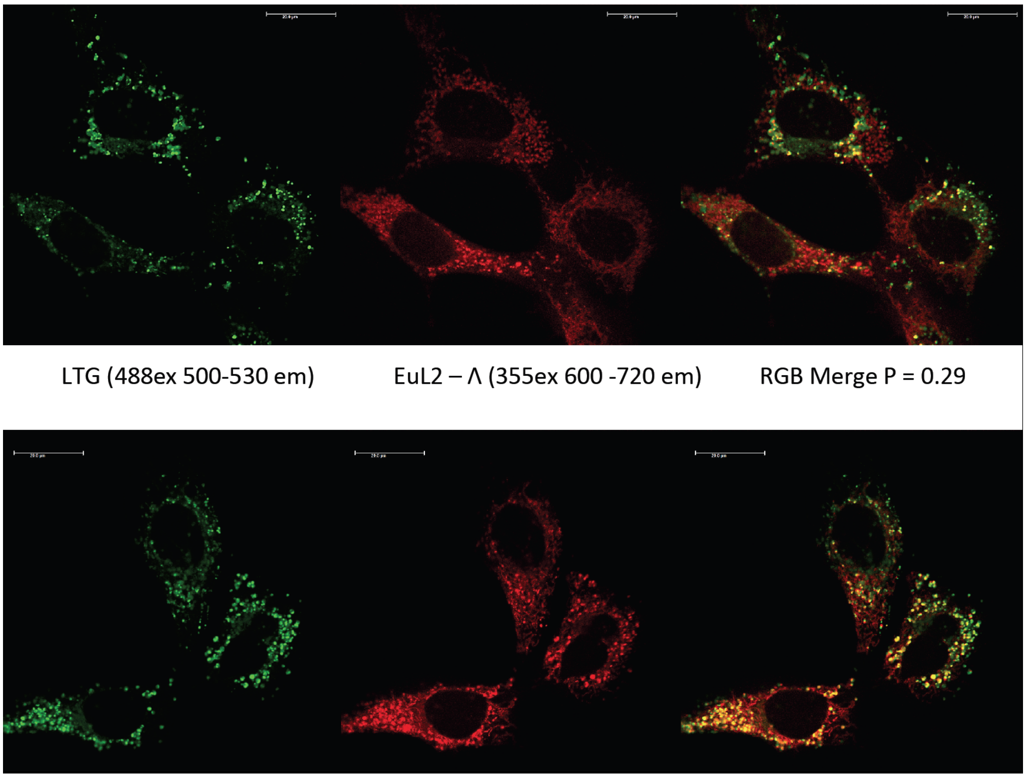

Figure S10 LSCM images of MCF-7 cells treated with $\mathit{\Lambda}$-**[EuL^2]** showing a poor correlation with the lysosomal stain , LysoTracker Green, P = 0.29 (24 h, 19 µM: Eu , λ_{exc} 355 nm, λ_{em} 605-720 nm; LTG: λ_{exc} 488 nm, λ_{em} 500-530 nm). Scale bars denote 20 µm.

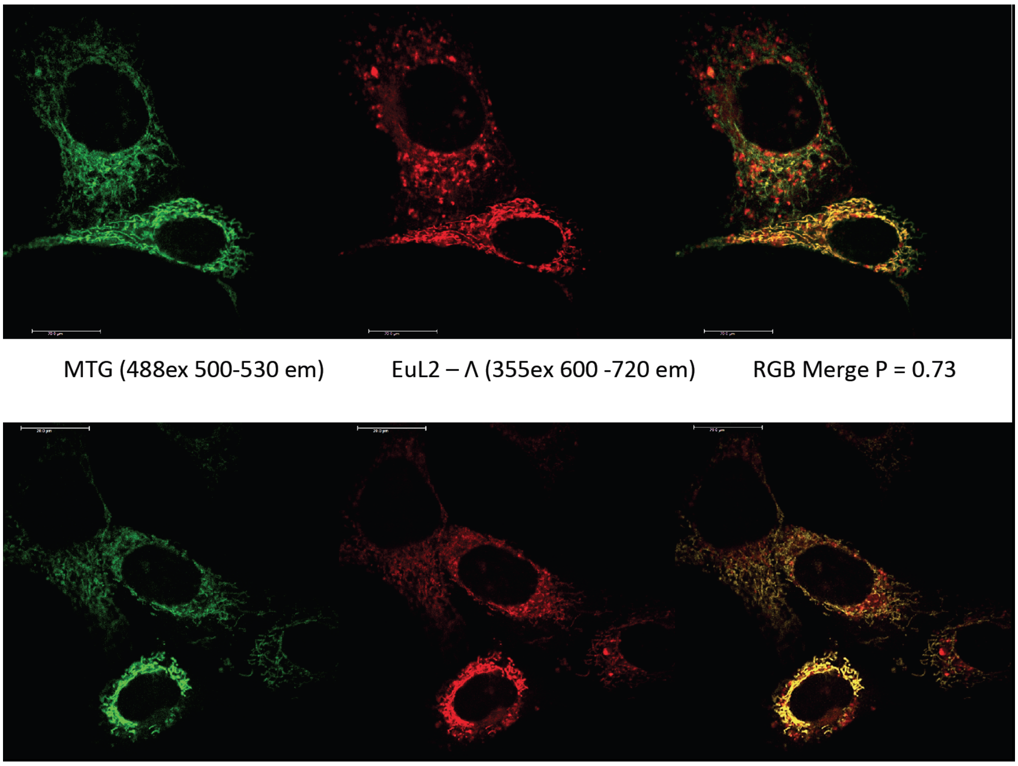

Figure S11 LSCM images of NIH 3T3 cells treated with $\mathit{\Lambda}$-**[EuL^2]** showing a good correlation with the mitochondrial stain , MitoTracker Green , P = 0.73 (24 h, 19 µM: Eu , λ_{exc} 355 nm, λ_{em} 605-720 nm; LTG: λ_{exc} 488 nm, λ_{em} 500-530 nm). Scale bars denote 20 µm.

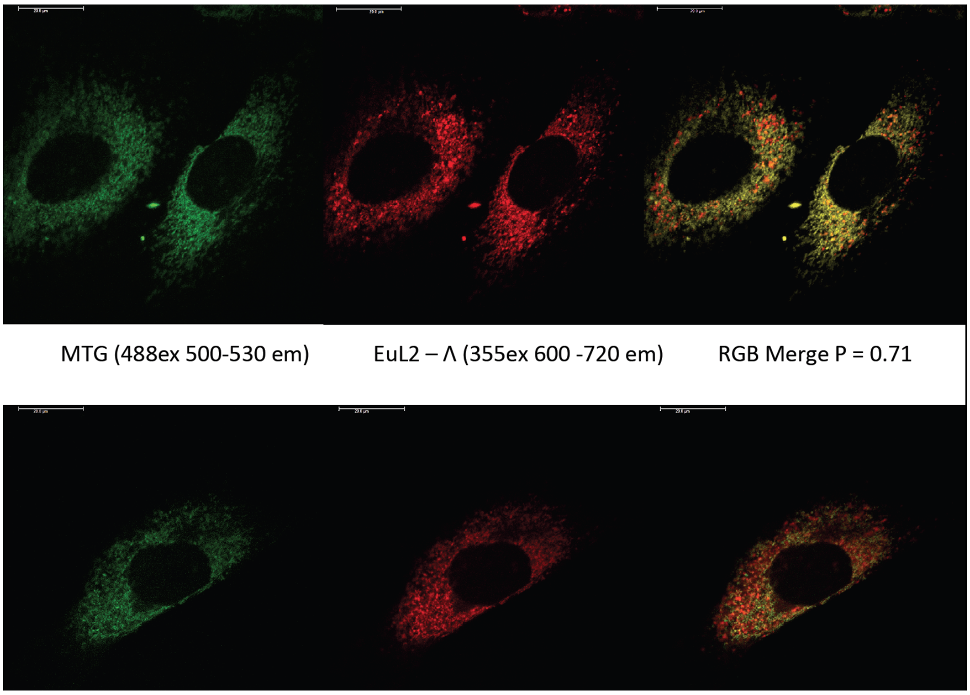

Figure S12 LSCM images of NIH 3T3 cells treated with Λ-**[EuL^2]** showing a good correlation with the mitochondrial stain , MitoTracker Green , P = 0.71 (24 h, 19 µM: Eu , λ_{exc} 355 nm, λ_{em} 605-720 nm; LTG: λ_{exc} 488 nm, λ_{em} 500-530 nm). Scale bars denote 20 µm

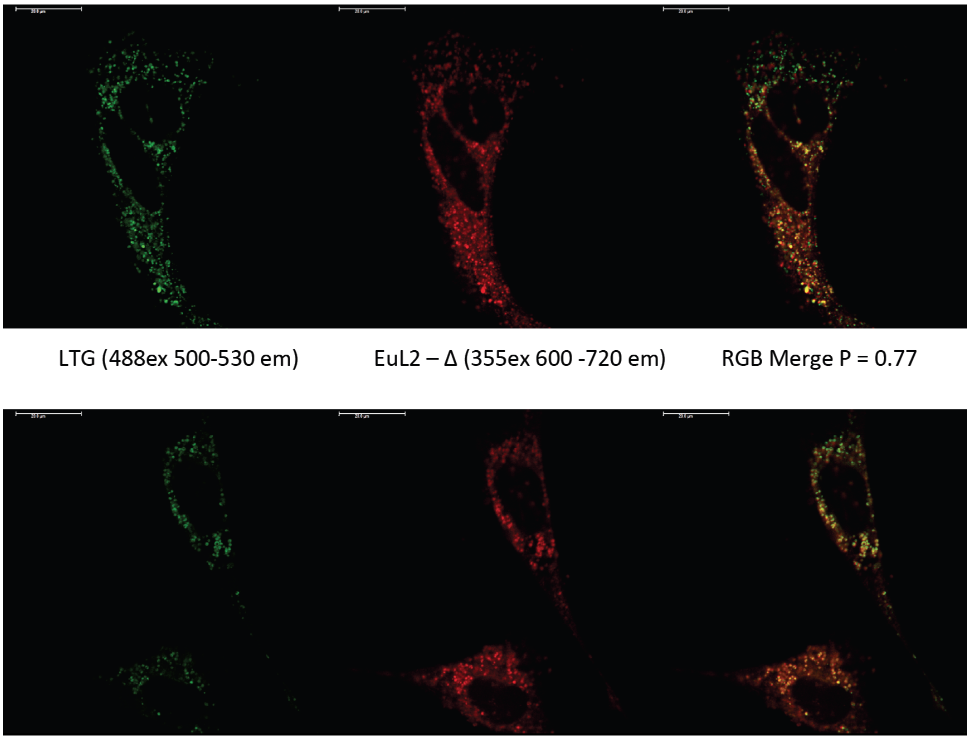

Figure S13 LSCM images of NIH 3T3 cells treated with Δ-**[EuL^2]** showing a good correlation with the lysosomal stain , LysoTracker Green , P = 0.77 (24 h, 19 µM: Eu , λ_{exc} 355 nm, λ_{em} 605-720 nm; LTG: λ_{exc} 488 nm, λ_{em} 500-530 nm). Scale bars denote 20 µm.
